# The Role of the Proteasome in Platelet Function

**DOI:** 10.3390/ijms22083999

**Published:** 2021-04-13

**Authors:** Abed El-Hakim El-Kadiry, Yahye Merhi

**Affiliations:** 1 Laboratory of Thrombosis and Hemostasis, Montreal Heart Institute, Research Centre, Montreal, QC H1T 1C8, Canada; abed.kadiry@gmail.com; 2 Biomedical Sciences Program, Faculty of Medicine, Université de Montréal, Montreal, QC H3T 1J4, Canada

**Keywords:** platelets, proteasome, NF-κB, atherothrombosis

## Abstract

Platelets are megakaryocyte-derived acellular fragments prepped to maintain primary hemostasis and thrombosis by preserving vascular integrity. Although they lack nuclei, platelets harbor functional genomic mediators that bolster platelet activity in a signal-specific manner by performing limited de novo protein synthesis. Furthermore, despite their limited protein synthesis, platelets are equipped with multiple protein degradation mechanisms, such as the proteasome. In nucleated cells, the functions of the proteasome are well established and primarily include proteostasis among a myriad of other signaling processes. However, the role of proteasome-mediated protein degradation in platelets remains elusive. In this review article, we recapitulate the developing literature on the functions of the proteasome in platelets, discussing its emerging regulatory role in platelet viability and function and highlighting how its functional coupling with the transcription factor NF-κB constitutes a novel potential therapeutic target in atherothrombotic diseases.

## 1. Introduction

Platelets are anucleate blood fragments originating from maturing megakaryocytes—precursor cells derived from pluripotent hematopoietic stem cells [1,2]. With a discoid shape at rest, a dimension of 3 μm × 0.5 μm, a lifespan of 10 days, and an average count of 250 million/mL of adult blood, platelets circulate in blood vessels, patrolling vascular endothelial cell lining [3,4]. Upon vascular damage or lesion, platelets adhere to exposed subendothelial matrix components, such as collagen, fibrinogen, and von Willebrand factor (VWF), thereby undergoing activation, secretion, spreading, and aggregation—unique platelet hemostatic and thrombotic functions that culminate in the conversion of αIIbβ3-bound fibrinogen to fibrin by thrombin, and the formation of a contractile fibrin-platelet plug that facilitates vascular recovery [5,6,7]. Besides primary hemostasis and thrombosis, the functions of platelets span inflammation, host defense, cancer, vascular tone regulation, and atherothrombotic diseases among others [8,9,10,11,12,13,14,15,16,17,18,19,20,21,22,23,24,25]. The execution of platelet function is met with a plethora of adhesive receptors (GPIb/IX/V complex, PSGL-1, GPVI immunoglobulin, α5β1/α2β1 integrins), activation receptors (protein tyrosine kinases, G-protein coupled receptors, αIIbβ3 integrin), secreted granule reservoirs (adhesion molecules, immunologic molecules, coagulation factors, chemokines, regulators of growth and angiogenesis, protease inhibitors, digestive enzymes, platelet agonists including ADP and Thromboxane A2, and platelet primers including epinephrine and soluble CD40L (sCD40L)), and dynamic cytoskeletal proteins (actin, myosin, spectrin) [26,27,28,29,30,31,32,33,34,35,36,37]. Generally, platelets inherit their cytoplasmic and membranous molecules from megakaryocytes early during platelet formation. However, platelets can perform limited de novo protein synthesis of important hemostatic and/or thrombo-inflammatory mediators, such as IL-1β and COX-1, in response to specific signals [27,38]. Deeper investigations into the biological intricacies of genome-devoid platelets have also revealed the presence of multiple transcription factors like nuclear factor-κB (NF-κB), translational machinery, microRNA, and more than 2500 mRNA transcripts [39,40,41,42,43,44,45,46,47,48,49,50,51,52]. These genomic apparatuses were demonstrated to fulfill genomic and/or non-genomic intra-platelet and/or extra-platelet functions [53,54,55,56,57,58,59,60,61]. Another functional paradox is that platelets are equipped with distinct and collaborative protein degradation mechanisms even though their protein synthesis capacity is limited. Their pool of degradative machinery groups calpain, caspases, matrix metalloproteinases, surface proteases, lysosomal proteases, and the proteasome [62]. In nucleated cells, proteasomal roles are well defined and primarily include the degradation of unneeded/misfolded proteins to maintain proteostasis, the regulation of signal transduction cascades, and the modulation of transcriptional activity (e.g., NF-κB activation) [63,64,65,66,67,68,69]. On the other hand, the role of the proteasome in platelets is less characterized. Herein, we orderly summarize the data generated hitherto on the regulatory roles of the proteasome in platelets and their contribution to platelet viability and function. We also highlight the importance of the proteasome/NF-κB dyad in platelets and its relevant therapeutic targeting in atherothrombotic diseases.

## 2. The Proteasome

### 2.1. Structures

**Figure 1:**
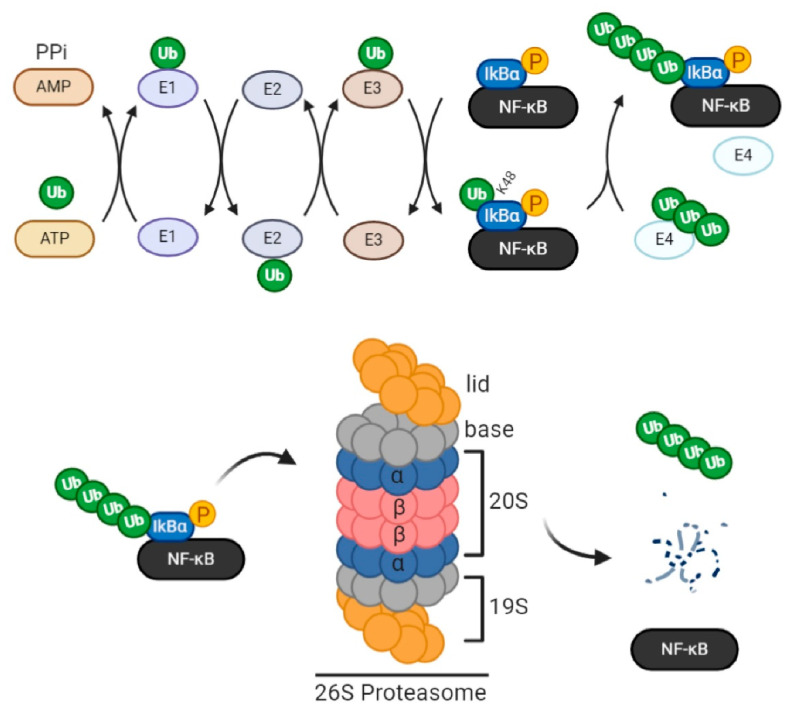
Components of the ubiquitin proteasome system (UPS). Protein ubiquitination is catalyzed sequentially by the ubiquitin-activating enzyme (E1), ubiquitin conjugation (E2) enzyme, and ubiquitin ligase (E3). First, the E1 enzyme binds and activates ubiquitin by consuming ATP. Then E2 forms an intermediate complex with activated ubiquitin. Lastly, E3 acts as a scaffold that interacts with both the E2-ubiquitin complex and the protein substrate, facilitating the transfer of ubiquitin from E2 to the substrate. An iso-peptide bond, thus, forms between the c-terminal glycine residue of ubiquitin and consensus lysine residues (7 total) of the substrate. Internal ubiquitin linkage at K11, 29, and 48 residues constitutes a tag for proteasomal trafficking. E4 ligase, a specific E3 subtype, can further elongate linear ubiquitin chains to create several polyubiquitin chain conformations. In this example, NF-κB is the protein substrate. Following its phosphorylation by IKK, IκB (inhibitory subunit of NF-κB) is polyubiquitinated for proteasomal trafficking. The standard proteasome 26S groups two regulatory subunits (19S each) comprising 2 multimers each, the lid and the base, in addition to a catalytic core (20S) whose β rings harbor caspase-, trypsin-, and chymotrypsin-like proteolytic activities (β1, β2, and β5, respectively). Upon substrate entry into the proteasome lid, the base facilitates the opening of the entry pore of 20S α rings, enabling protein translocation and degradation in the core by β rings. Furthermore, the base, due to the chaperone-like activity of its highly specific ubiquitin receptor subunits, ensures the selectivity of degradation, as it permits the entry of ubiquitinated proteins only, by forming covalent bonds with monomeric or polymeric ubiquitin chains. The 19S subunit also contains deubiquitinating enzymes (DUBs) that facilitate protein unfolding, translocation, and degradation within the core. In the given scenario, IκB is deubiquitinated and degraded within the proteasome so that activated NF-κB becomes free to function.

The standard proteasome is a 26S multi-catalytic protease complex (2.4 MDa) comprising two 19S regulatory caps (750 kDa each) and a central 20S proteolytic subunit (around 750 kDa) found within the core. The 19S complex is an arrangement of 2 multimers, the lid, and the base. The lid comprises up to 10 non-ATPase subunits. The base comprises 2 non-ATPase subunits and 6 homologous hexamer ring-forming ATPases (PSMC2/1/4/6/3/5) and is responsible for the assembly of the 19S regulatory particles with the 20S core particle, allowing the formation and activation of the ATP-dependent 26S proteasome. The 20S core particle is composed of 28 heterogeneous subunits arranged in 4 rings: 2 beta rings on the inner side and 2 pore-forming non-catalytic alpha rings on the outer side. Each ring thus groups 7 subunits (β1–7 and α1–7). Among the beta subunits, 3 perform caspase-, trypsin-, and chymotrypsin-like proteolytic activities (β1/PSMB6, β2/PSMB7, and β5/PSMB5, respectively) by cleaving peptide bonds following acidic, basic, and hydrophobic amino acids, respectively. Alone, the proteolytic 20S core performs ubiquitin-independent degradation. Assembled with the 19S regulatory particles to form the 26S proteasome, it specifically degrades ubiquitin-conjugated proteins in an ATP-dependent manner (Figure 1).

Ubiquitin is a eukaryotic sequence of 76 amino acids weighing approximately 8 kDa. Its coupling to proteins is catalyzed sequentially by the ubiquitin-activating (E1) enzyme, the ubiquitin conjugation (E2) enzyme, and the ubiquitin ligase (E3). First, in the reaction, the E1 enzyme binds and activates ubiquitin by consuming ATP. Then E2 forms an intermediate complex with activated ubiquitin. Lastly, E3 acts as a scaffold that interacts with both the E2-ubiquitin complex and the protein substrate, facilitating the transfer of ubiquitin from E2 to the substrate. An iso-peptide bond thus forms between the c-terminal glycine residue of ubiquitin and the consensus lysine residues in the substrate. Noteworthy, a specific E3 subtype called E4 ligase can further elongate linear ubiquitin chains at internal lysine residues to create several polyubiquitin chain conformations. This internal ubiquitin linkage serves as a code that determines the protein’s fate. For instance, internal ubiquitin linkage at lysine 11, 29, and 48 residues is a protein tag for proteasomal degradation. Linkages at the other 4 residues (K6, K27, K33, K63) modulate protein activity, localization, or interaction/scaffolding. Along with the proteasome, ubiquitin and its enzymes form the ubiquitin-proteasome system (UPS) (Figure 1) [70,71,72,73,74,75].

More in-depth, during 26S proteasome formation and activation, PSMC1 facilitates the opening of the entry pore of 20S α subunits, enabling protein translocation and degradation in the core. Meanwhile, the base, with the chaperone-like activity of its highly specific ubiquitin receptor subunits, further ensures the selectivity of the process, permitting the entry of ubiquitinated proteins only, by forming covalent bonds with monomeric or polymeric ubiquitin chains. In addition to its substrate recognition, unfolding, and protein translocation properties, the 19S regulatory subunit conducts deubiquitination [63,67,76,77]. In general, deubiquitination is carried by deubiquitinating enzymes (DUBs) and can be UPS-independent; therein, DUBs alter protein trafficking or enhance protein stability. Around 80 DUBs have been identified and grouped into 6 families based on the homology of their catalytic domain sequences. They exist either as free enzymes or are associated with large enzyme complexes, such as the proteasome. In UPS, deubiquitination is mediated by three 19S-associated DUBs (USP14, UCHL5, and Rpn11) and facilitates the unfolding, translocation, and degradation of proteins within the 20S core [78,79].

In addition to its standard form, the proteasome exists in other forms, such as the thymoproteasome (TPr), intermediate proteasomes, hybrid proteasomes, and the immunoproteasome (IP) [80,81]. For example, the IP is responsible for MHC class I-mediated antigen presentation through generating antigenic peptides. While the IP is constitutively expressed in immune cells of myeloid or lymphoid origins, it is synthesized in response to interferons or lipopolysaccharides in nucleated cells. IP assembly occurs through the substitution of proteolytic β1, β2, and β5 subunits of the 20S core with their IP counterparts: β1i/PSMB9, β2i/PSMB10, and β5i/PSMB8. In contrast to the 26S proteasome, which harbors 19S regulatory subunits, the immunoproteasome is regulated by two heptameric 11S subunits, which consist of PA28 α/PSME1 and β/PSME2 subunits and are induced by interferon-γ [82,83] to facilitate substrate access into the proteasomal core [84,85].

### 2.2. Functions

The functional importance of proteasome-mediated protein degradation was initially delineated through in vivo examination of UPS characteristics in pathological conditions. In neurodegenerative diseases, for instance, UPS was found to be impaired [86] with a widespread accumulation of ubiquitinated proteins [87]. In a subclass of Parkinson’s disease, E3 ligase was reported to be mutated [88]. In age-related disorders, a decline in proteasomal activity and an accumulation of misfolded proteins were also recorded [89]. In humans, PSMB8 mutations cause a rare genetic disorder (chronic atypical neutrophilic dermatosis with lipodystrophy and elevated temperature, or CANDLE) that presents with accumulated ubiquitinated proteins, high levels of type I interferon, and thrombocytopenia [90].

To better assess the roles of the proteasome in cellular functions, genetic and pharmacological methodologies were approached. A study by Bedford et al. [77] showed that PSMC1 deletion in vivo is embryonically lethal, and neuronal 26S proteasome depletion leads to neurodegeneration. Disrupting the ubiquitin gene also causes neuronal death and impairs energy expenditure systems, exposing the organism to obesity [86]. The extensive use of multiple natural or synthesized proteasome inhibitors (Table 1) including epoxomicin, lactacystin, DCI, HgCl_2_, bortezomib (PS-341), carfilzomib, MG132, PCMB, PSI (benzyloxycarbonyl-Ile-Glu(O-tert.-butyl)-Ala-leucinal), and Mersalyl acid has ascribed to the proteasome multiple cellular roles [91,92,93,94,95,96,97]. Therefrom, it was found that proteasome-mediated protein degradation/processing not only removes aberrant proteins but also regulates (1) signal transduction cascades, (2) receptor turnover/desensitization, (3) enzymatic activity, (4) cell cycle progression, (5) cell growth/differentiation, (6) survival/apoptosis, (7) gene transcription/repair, (8) antigen presentation (IP-specific), (9) oxidative stress-induced cellular damage (IP-specific), and (10) cellular maturation (TPr-specific) [63,64,65,66,67,68,69,98,99,100].

## 3. The Platelet Proteasome

Yukawa et al. [101] were the first to purify the platelet proteasome with a chromatography column, initially reporting the presence of several subunits with chymotrypsin- and trypsin-like activities. Subsequent investigations corroborated that platelets express (1) the standard proteasome (20S and 26S) and the IP [62,82,102,103], (2) functional mono and polyubiquitination systems [104,105], (3) UPS-related mRNAs [52], and (4) DUBs [106]. Still and all, the role and modulation of proteasome-mediated protein degradation in these anucleate cell fragments remain elusive.

### 3.1. Intra- and Extra-Platelet Activators and Regulators

The platelet proteasome is constitutively active, yet multiple molecules augment its activity [69]. At relatively high concentrations, poly-lysine and SDS are considered proteasome activators. Several other natural and synthetic lipid- and peptide-based molecules are known to induce proteasomal activation as well, even at low concentrations [107]. Platelet agonists including collagen and thrombin induce, respectively, a 10- and 7-fold increase in proteasomal chymotrypsin-like activity [69]; ADP also causes the proteasomal activity to double [108]. Platelet receptors including toll-like receptor 4 (TLR4) (lipopolysaccharides-ligated), PAR1 (thrombin-ligated), GPIb-IX-V (thrombin- and VWF-ligated), and P2Y_12_ (ADP-ligated) communicate with the proteasome [104]. In terms of intraplatelet regulation, the activation of the proteolytic machinery proceeds through cAMP production and is PKA-dependent [109]. ATP, Mg^2+^, PLCγ pathway, and calcium-dependent effectors like calpain and PKC also regulate proteasomal enzymatic activity [69,101,110,111,112]. Yukawa et al. [101] purified form platelets an endogenous polypeptide complex (170 kDa) that potentiates and dose-dependently enhances proteasomal chymotrypsin- and trypsin-like activities. The same group then characterized the latter 170-kDa polypeptide complex, demonstrating that it functions as a positive allosteric effector [113]. Ostrowska et al. [102] later discovered that platelets also express functional PA28 (PSME1) that controls antigen processing through stimulating proteasomal chymotryptic-like activity.

### 3.2. Roles

As in nucleated cells, platelet proteasome performs chymotrypsin-, trypsin-, and caspase-like proteolytic activities which largely ascribe to the proteasome a regulatory role in platelet production, viability, and function [73].

#### 3.2.1. In Platelet Production and Viability

The proteasome modulates platelet production and lifespan. This is evidenced in that proteasome inhibition reduces platelet count and half-life by 50%. Simultaneously, changes in apoptotic markers (e.g., phosphatidylserine (PS) exposure, pro-apoptotic BAX protein upregulation, mitochondrial transmembrane potential decrease) and macrophage-mediated clearance are observed [53,114]. Bortezomib, the first proteasome inhibitor to hit clinical practice as a second-line treatment for multiple myeloma [115], also induces thrombocytopenia in patients by influencing the production of platelets from megakaryocytes. Specifically, bortezomib use elevates the levels of activated small GTPase Rho, a negative regulator of platelet formation [116,117,118]. In addition, after bortezomib withdrawal, drug-associated thrombotic microangiopathy is resolved in patients [119]. These data suggest that the proteasome is involved in physiologic and pathophysiologic platelet states, overall regulating platelet thrombopoiesis and viability [120].

#### 3.2.2. In Platelet Function

##### Pathological Findings

Examining the characteristics of the platelet proteasome in pathological settings allows us to better understand its degree of contribution to platelet function. For instance, *E. coli*-induced sepsis upregulates platelet PA28 on mRNA and protein levels and augments proteasomal proteolytic activity [121]. In hemolytic conditions, the increased levels of oxidative stress in platelets are associated with (1) reduced proteasomal activity and augmented protein ubiquitination, (2) increased BAX levels, and (3) premature death [122]. In coronary artery disease (CAD), patients present with decreased expression of PSMB8 [123]. In patients with ANKRD26 gene-related thrombocytopenia, proteasome-studded particulate cytoplasmic structures are observed in platelets, which additionally show reduced aggregation responses [124]. However, whether these UPS characteristics are causing factors or consequences of such pathologies requires further investigation.

##### Pharmacological Findings

A clearer insight into the impact of the proteasome on platelet activation was obtained by pharmacologically targeting the UPS components, primarily the standard proteasome and the proteasome-associated DUBs.

##### Proteasome Inhibitors

Dupré et al. [64] showed using PSI that the proteasome desensitizes platelets to the platelet-activating factor (PAF), a potent phospholipid mediator associated with multiple inflammatory diseases, via downregulating the ubiquitin-coupled PAF receptor upon ligand stimulation. Another negative regulatory role for the proteasome in platelet activation was reported. Using MG132, it was shown that the proteasome can degrade platelet CD36, a receptor for oxLDL and cell-derived microparticles [125]. Besides, platelet treatment with bortezomib was shown to attenuate VASP phosphorylation—an inhibitory signaling pathway that reduces P-selectin exposure and fibrinogen binding to the αIIbβ3 integrin receptor—thereby enhancing platelet aggregation [126,127].

**Figure 2:**
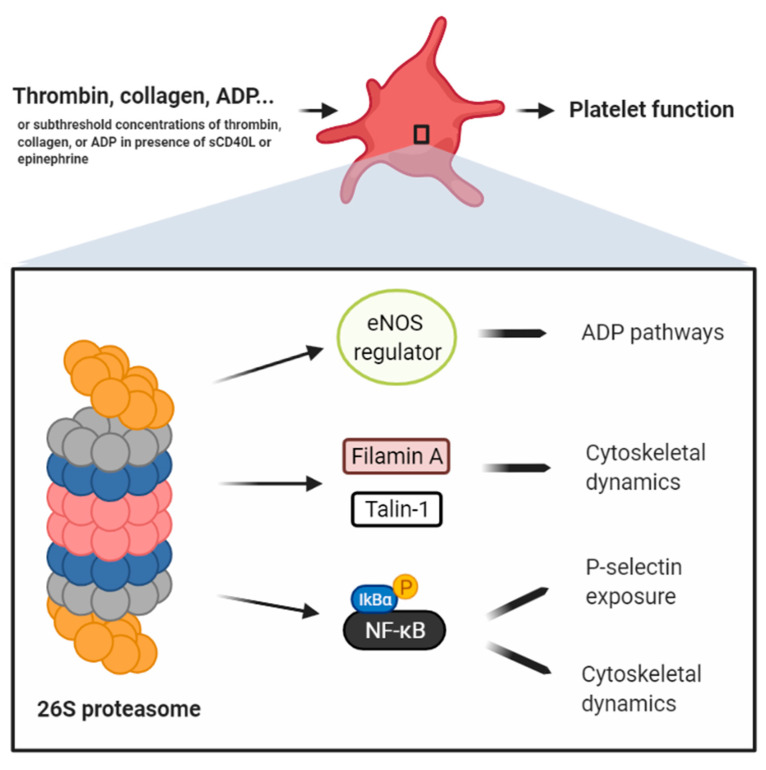
Schema compiling the data of different reports ascribing to the proteasome a positive regulatory role in platelets. Avcu et al. [129] claim that the platelet proteasome degrades eNOS regulators, thereby preventing the production of NO, an ADP receptor antagonist. Gupta et al. [104] showed that the proteasome, in response to the most potent platelet agonist thrombin, only partially regulates platelet function via the cleavage of cytoskeletal regulators, such as Filamin A and Talin-1. Karim et al. [131] reported that the platelet proteasome, in response to collagen or thrombin, only partially regulates platelet activation, P-selectin exposure, and aggregation, through the activation of NF-κB. Grundler et al. [132] showed that the platelet proteasome, in response to collagen and via NF-κB activation, only partially regulates platelet aggregation. Our group has shown that sCD40L primes platelets, but does not induce their aggregation, by activating platelet NF-κB and inducing P-selectin exposure and dynamic cytoskeletal shape changes [133,134]. sCD40L/NF-kB mediate platelet activation and aggregation in response to low doses of thrombin or collagen [134]. Although further validation is required, the sum of these data corroborates that the platelet proteasome/NF-κB dyad is a positive regulator of platelet priming, in which P-selectin exposure and dynamic cytoskeletal shape changes are enhanced. This intermediate platelet state eventually potentiates platelet activation and aggregation when additional subthreshold concentrations of platelet agonists are secured.

Contrastingly, most other reports corroborate a positive regulatory role for the proteasome in platelet activation (Figure 2). Using PSI and MG132 in megakaryocytes, Mitchell et al. [128] showed that the biogenesis of αIIbβ3 integrin is regulated by the proteasome through the degradation of misfolded pro-αIIb subunits. Although contravened by Koessler et al. [108,126] who used different platelet agonist doses, bortezomib use conferred upon the proteolytic complex a role in collagen-mediated ATP release and ADP-induced platelet aggregation—the latter function being established by degrading eNOS regulators and preventing the production of NO, an ADP receptor antagonist [129]. Besides ameliorating endothelial function and blocking NF-κB activation in vascular and blood cells, this might explain the reduction of thromboembolic events in vivo upon proteasomal antagonism [130]. Furthermore, administering MG132 to mice, or treating their platelets with MG132 and reinjecting them, delays induced occlusive thrombosis. In vitro, this is attributed to the inhibition of cleavage of cytoskeleton regulators, such as Filamin A, which links GPIb-IX-V complex to cytoskeletal actin filaments, and Talin-1, which plays a role in platelet spreading. As such, both proteins accumulate in the cytoplasm, ubiquitinated and in their native non-cleaved form, overall causing reduced platelet (1) adherence and spreading, (2) surface release of PS-expressing prothrombotic microparticles, (3) aggregation, and (4) fibrin-platelet clot retraction, as compared to a positive control (platelets stimulated with subthreshold-dose thrombin) [104]. Likewise, Karim et al. [131] reported decreased (1) αIIbβ3 activation, (2) P-selectin and PS exposure (markers of platelet degranulation and procoagulant activity), (3) intracellular calcium levels, and (4) aggregation in platelets pretreated with MG132 then stimulated with subthreshold doses of collagen and/or thrombin. Noteworthy, both studies demonstrate that the levels of platelet activation markers and platelet aggregation are unaffected by proteasomal antagonism in response to higher doses of thrombin (above 0.05 U/mL). This suggests weaker ties between the proteasome and thrombin’s high-affinity receptor (PAR1) as compared to the other thrombin receptor GPIb-IX-V [104,131].

Few other studies argue that the inhibition of platelet aggregation using proteasome inhibitors does not proceed through proteasome antagonism but rather and particularly through NF-κB inhibition [135]. This observation might be explained by the fact that collagen-mediated platelet activation through the GPVI receptor is transduced through spleen tyrosine kinase (Syk) that phosphorylates several adaptor proteins in the pathway. Indeed, upon GPVI ligation, Syk is ubiquitinated but not degraded; its activity becomes 5-fold higher, after which it is thought to modulate platelet function. Likewise, collagen-related peptides were demonstrated to elevate the amount of ubiquitinated proteins in platelets for signaling purposes [105,136]. Another study showed no effect on P-selectin exposure upon proteasome inhibition [69].

Overall, the scarce data available so far suggest that the most potent platelet activation pathways are either upstream or work in parallel with the proteasome. Further studies might therefore warrant a clearer link between the proteasome and platelet activation and aggregation.

##### DUB Inhibitors

As aforementioned, proteasome-associated DUBs are essential for proteasomal functioning. Henceforth, in a similar manner to proteasome antagonists, proteasome-associated DUB antagonists have accredited a positive regulatory role for the proteasome in platelet activation. Gupta et al. [106] showed that treating platelets with b-AP15, a specific inhibitor of USP14 and UCHL5, significantly reduces αIIbβ3 activation and platelet aggregation in response to multiple platelet agonists. Interestingly, this study used high doses of agonists (0.2 U/mL, 2 μg/mL, and 5 µM of thrombin, collagen, and ADP, respectively), thereby opposing previous reports showing no effect with MG132 or bortezomib pretreatment on the aggregation of platelets also stimulated with high-dose thrombin (above 0.05 U/mL) [104,131] or collagen (5 μg/mL) [126] or on the aggregation of platelet-rich plasma (PRP) stimulated with collagen (10 μg/mL) or ADP (10 µM) [108]. Additionally, b-AP15 pretreatment diminished P-selectin exposure [106], another observation unreported with PSI- [69] or MG132-treated platelets [104,131]. These discrepancies between the data of both types of UPS antagonists might be resolved with further investigation.

Other players in UPS like E3 ligases are also reported to regulate platelet activation and signal transduction pathways through different mechanisms that were recently reviewed, along with data on the platelet IP, elsewhere [103].

## 4. NF-κB/Proteasome Coupling in Platelets

NF-κB is a family of cytoplasmic proteins that exist as dimers formed from five DNA-binding subunits: p50 (NF-κB1), p65 (RelA), cRel, p52 (NFκB2), and Rel B. In its canonical pathway, NF-κB is initially induced upon ligation of multiple receptors including tumor necrosis factor receptors, toll-like receptors, and B-cell receptors, which activate transforming growth factor β-activated kinase 1 (TAK1). In this pathway, NF-κB dimers are found inactivated by the inhibitory subunit IκB (IκBα or IκBβ, with IκBα being the most prevalent). The non-canonical pathway is triggered by several receptors including the lymphotoxin-β receptor and the B-cell activating factor receptor, which activate the NF-κB-inducing kinase (NIK). In this pathway, NF-κB subunits associate with p100.

Generally, NF-κB activation requires the IκB kinase (IKK). In the canonical pathway, TAK1 activates IKK, which phosphorylates IκB, causing its ubiquitination and degradation by the proteasome (Figure 1). In the non-canonical pathway, NIK activates IKK, which phosphorylates p100, causing its ubiquitination and processing into p52 by the proteasome. Following their ubiquitination and proteasome-mediated activation, NF-κB dimers translocate into the nucleus, of mostly immune cells, where they bind DNA and regulate inflammatory, survival, proliferation, differentiation, transmigration, chemotaxis, and defense genes [137,138,139]. In fact, the genomic roles of NF-κB transcription factors in immune cells are well characterized since their discovery in B lymphocytes more than 30 years ago [140,141,142,143,144]. Notable, almost all NF-κB subunits are ubiquitinated by E3 ligases, and their ubiquitination serves as a tag not only for proteasomal degradation/processing to eventually allow the control of gene expression but also for performing other proteasome-independent functions outside the scope of this review article [145,146].

Platelets also express NF-κB, which seems to function mainly in non-genomic ways [42,133,134,147,148,149,150,151,152,153]. Our recent extensive review article employs platelet NF-κB as its primary focus, showcasing its role in positively regulating platelet survival, priming, activation, and aggregation as well its potential extra-platelet role following cellular endocytosis of NF-κB-engulfing platelet microparticles [53].

In platelet function, proteasome/NF-κB coupling is important—a realization evident in that NF-κB activation necessitates the proteasome and that proteasome inhibitors are also NF-κB inhibitors (Table 1, Figure 1 and Figure 2). For instance, PSI was demonstrated to inhibit collagen-induced platelet aggregation not necessarily due to inhibiting all proteasome functions [135] but specifically NF-κB activity [132]. Additionally, epoxomicin treatment of PRP was shown to mitigate the aggregation of collagen-stimulated platelets in an NF-κB dependent manner [132]. Our previous data on the functions of the platelet primer, sCD40L, show that sCD40L activates platelet NF-κB, inducing granular secretion and so P-selectin translocation to the platelet surface [133]. We have also shown that in presence of low doses of platelet agonists, such as thrombin and collagen, sCD40L potentiates platelet aggregation through NF-κB activation [134]. Our most recent data validate the involvement of the proteasome/NF-κB dyad in the platelet priming functions of sCD40L (potentiation of platelet aggregation and fibrin-platelet clot formation) in presence of subthreshold doses of collagen and thrombin [154]. Taken together with various other reports showing that proteasome inhibition reduces platelet aggregation and/or secretion only in presence of low doses of platelet agonists [69,104,108,126,131,149], it can be speculated that the proteasome/NF-κB dyad does not regulate platelet activation but rather platelet priming, in which platelets are not fully activated to aggregate (no Ca2+ influx or strong αIIbβ3 activation) but present with activation markers including P-selectin exposure and dynamic cytoskeletal shape changes [155,156,157]. Eventually, platelet priming potentiates platelet activation and aggregation when low doses of platelet agonists are secured [53,133,134,156,158,159].

In a pathological context, high plasma levels of platelet primers are associated with atherothrombotic disorders, such as CAD and cerebrovascular disease, which entail the highest mortality rate among the 2nd runner in death causes in Canada—cardiovascular diseases [160,161]. Additionally, platelet primers correlate with resistance to antiplatelet therapy like aspirin (ASA) [157,162,163,164,165,166]. Indeed, we have recently shown that ASA does not affect platelet NF-κB signaling induced by sCD40L alone [159]. On a similar note, the NF-κB/proteasome dyad orchestrates chronic inflammatory diseases, such as atherothrombosis, by targeting various genes (cytokines, chemokines, immune receptors, coagulation regulators, anti-apoptotic molecules, adhesion molecules, cell cycle regulators, etc.) in thrombo-inflammatory mediators including endothelial cells, monocytes, neutrophils, lymphocytes, and platelets [53,167,168]. Platelets are, indeed, among the principal mediators of atherothrombotic diseases due to their adherence to atherosclerotic plaques and subsequent hyperactivation and secretion of multiple pro-inflammatory molecules including adhesive factors (fibrinogen, fibronectin, VWF, P-selectin), coagulation factors (factor V, factor XI, plasminogen, protein S), growth factors (platelet-derived growth factor, epidermal growth factor, basic fibroblast growth factor), and chemokines/cytokines (IL-1β, sCD40L) [15,16,17,169,170,171,172,173,174,175,176,177,178,179]. Therefore, the NF-κB/proteasome dyad might maneuver the transition of platelet phenotype into chronic pro-inflammatory blood entities. Henceforth, targeting the NF-κB/proteasome dyad in platelets by proteasome inhibition might be therapeutically beneficial in atherothrombotic diseases, given the protective effects reported upon targeting the same dyad in other settings including myocardial infarction [180,181], stroke [182], cancer [183], hypertensive injury [184], and organ transplantation [185,186].

## 5. Conclusions

Despite their anucleate nature and hand-me-down proteins from megakaryocyte precursors, platelets comprise genomic mediators like NF-κB and protein degradation machinery like the proteasome. The few studies available thus far on platelet proteasome show that it regulates platelet lifespan and viability yet only partially enhances platelet activation and aggregation by activating NF-κB. In this context, the NF-κB/proteasome dyad could be more pertinent in platelet priming, an intermediate state that predisposes platelets to pronounced activation and aggregation and correlates with atherothrombotic diseases and resistance to antiplatelet therapy. Therefore, targeting the NF-κB/proteasome axis in platelets might assist in contriving novel pharmacological compounds for the treatment of atherothrombosis.

## Figures and Tables

**Table 1:** Some proteasome antagonists and their characteristics.

Inhibitor	Properties	Proteasomal Binding and Targeted Activities	Other Cellular Effects
**Bortezomib**	First-class; FDA-approved (Velcade^®^) for first-line treatment of multiple myeloma	Reversibly binds 26S proteasome and immunoproteasome; chymotrypsin -> caspase -> trypsin-like activity	NF-κB inhibition; cell apoptosis due to accumulation of proteins, stress induction, and disruption of cell cycle
**Carfilzomib**	New generation; FDA-approved (Kyprolis^®^) against relapsing multiple myeloma; less toxic than Bortezomib	Irreversibly binds 20S proteasome and immunoproteasome; chymotrypsin-like activity	Cell apoptosis
**Lactacystin**	Isolated from soil Actinomycetes; Prodrug, metabolized into a β-lactone (Omuralide) in vivo; inhibits non-proteasome proteases like cathepsin	Irreversibly binds 20S proteasome and immunoproteasome; all activities, with preference to chymotrypsin-like activity	Inhibits cellular growth; cell apoptosis; NF-κB downregulation
**Epoxomicin**	Isolated from Actinomycetes strain; specific	Irreversible binds 20S proteasome; all activities, with preference to chymotrypsin-like activity	Inhibits NF-κB signaling
**MG132**	Peptide aldehyde isolated from Chinese medicinal herbs; first choice to study UPS in human cell lines	Irreversibly binds 20S proteasome; all activities, with preference to chymotrypsin-like activity	Cell cycle arrest and apoptosis; inhibits NF-κB activation

## Abbreviations

ASA, aspirin; CAD, coronary artery disease; CD40L, CD40 ligand; DUB(s), deubiquitinating enzyme(s); IKK, IκB kinase; NF-κB, nuclear factor-κB; NIK, NF-κB-inducing kinase; PAF, platelet activating factor; PRP, platelet-rich plasma; PS, phosphatidylserine; sCD40L, soluble CD40 ligand; TAK1, transforming growth factor β-activated kinase 1; TLR4, toll-like receptor 4; UPS, ubiquitin proteasome system; VWF, von Willebrand factor.
